# Monitoring of Water Spectral Pattern Reveals Differences in Probiotics Growth When Used for Rapid Bacteria Selection

**DOI:** 10.1371/journal.pone.0130698

**Published:** 2015-07-02

**Authors:** Aleksandar Slavchev, Zoltan Kovacs, Haruki Koshiba, Airi Nagai, György Bázár, Albert Krastanov, Yousuke Kubota, Roumiana Tsenkova

**Affiliations:** 1 Kobe University, Graduate School of Agricultural Science, Biomeasurement Technology Laboratory, 1–1 Rokkodai, Nada-ku, Kobe 657–8501, Japan; 2 University of Food Technologies, Department of Microbiology, 26 “Maritza” Blvd., 4002 Plovdiv, Bulgaria; 3 Corvinus University of Budapest, Faculty of Food Science, Department of Physics and Control, 14–16 Somlói str., Budapest 1118, Hungary; 4 Kaposvár University, Faculty of Agricultural and Environmental Sciences, Institute of Food and Agricultural Product Qualification, 40 Guba Sándor str., Kaposvár 7400, Hungary; 5 University of Food Technologies, Department Biotechnology, 26 “Maritza” Blvd., 4002 Plovdiv, Bulgaria; Agricultural University of Athens, GREECE

## Abstract

Development of efficient screening method coupled with cell functionality evaluation is highly needed in contemporary microbiology. The presented novel concept and fast non-destructive method brings in to play the water spectral pattern of the solution as a molecular fingerprint of the cell culture system. To elucidate the concept, NIR spectroscopy with Aquaphotomics were applied to monitor the growth of sixteen *Lactobacillus bulgaricus* one *Lactobacillus pentosus* and one *Lactobacillus gasseri* bacteria strains. Their growth rate, maximal optical density, low pH and bile tolerances were measured and further used as a reference data for analysis of the simultaneously acquired spectral data. The acquired spectral data in the region of 1100-1850nm was subjected to various multivariate data analyses – PCA, OPLS-DA, PLSR. The results showed high accuracy of bacteria strains classification according to their probiotic strength. Most informative spectral fingerprints covered the first overtone of water, emphasizing the relation of water molecular system to cell functionality.

## Introduction

Probiotic bacteria are non-pathogenic microorganisms that, when ingested in sufficient viable numbers, exert a positive influence on the host[1]. Some of the beneficial effects of probiotics include balancing of the gastro-intestinal (GI) tract microflora, improvement of immune response[2], production and improvement of the utilization of nutrients[3], decrease in the symptoms of lactose intolerance and allergies in susceptible individuals[4], reduction of the risk of cancer[5], alleviation of irritable bowel syndrome and inflammatory bowel diseases[6,7]. The mechanism of probiotic activity has not been established yet, but it probably includes modification of GI tract pH levels[8], pathogens antagonism through the production of antimicrobials[9], competition for receptor sites[10], nutrients and growth factors, stimulation of immune cells[11] and lactase production[12].

In order to exert their positive effect the beneficial bacteria must reach the colon in relatively high viable cell concentrations[13]. They must survive the transit through the stomach and the small intestines, where they are exposed to harsh conditions, such as low pH values and pepsin presence and high bile salt concentrations[14]. Thus, the most important probiotic characteristic is the ability of surviving the harsh environment in the upper gastro-intestinal tract. Another key feature is the production of sufficient amount of biomass during the cultivation process in the production facilities. Therefore, strains possessing high growth rates and capable of gaining high amount of biomass in a short period are more suitable for industrial production of probiotics and probiotic foods.

A major issue in the production of probiotics and probiotic functional foods is the selection of strains exhibiting strong probiotic characteristics in each respective environment. Currently, two main strategies have been applied for the selection of probiotic strains: selection of strains with particular genes and *in vitro* examination of strain growth under model conditions of the digestive tract[14,15]. These methods are time-consuming, require expensive equipment and consumables, and they give uncertain results. However, a quick and inexpensive method, which allows rapid, *in vivo* comprehensive bacteria efficiency evaluation, is needed.

In recent years, the new approach of “aquaphotomics” has been proposed[16]. It is based on dynamic spectroscopy of the water molecular system of the examined biological system, using its water spectrum as a molecular mirror[16,17] that reflects the rest of the solution. The spectrum contains a big amount of information about the target object, coded by the water molecular arrangement. When bacteria growth is monitored by its NIR spectra, huge amount of data is obtained. Further on, in Aquaphotomics, to extract all the information hidden in the spectra and related to the specificity of each strain, different multivariate statistical methods are applied.

This new approach has been successfully applied in the research and diagnostics of various species[17–20] and for identification and discrimination of bacterial species at very low concentration. It has been proven that extracellular metabolites played more significant role in successful spectral qualitative model performance[21].

Aquaphotomics using near infrared (NIR) spectroscopy is time-efficient and it allows rapid, chemical-free, non-invasive *in vivo* assessment, provides an opportunity for researching live microorganisms in the cultivation process[19,20]. The method is very sensitive to even traces of analytes. This makes it of first choice when the target components important for the characterization of the studied systems affect the water structure and are presented in very low concentrations[17].

Aquaphotomics studies the biological systems as whole entities in a holistic way and presents a new and unique point of view regarding their functionality. Water spectral patterns of the living microorganisms present information about their functionality and could serve as fingerprints of cells phenotype. Therefore, replacing the phenotypic and genetic approach for probiotic bacteria selection with Aquaphotomics is an innovative strategy. Thus the goal of this research was to evaluate the application possibilities of Aquaphotomics in rapid selection and evaluation of bacterial strains possessing different probiotic properties.

## Methods

### Bacterial strains

Seven probiotic and eleven non-probiotic strains (genus *Lactobacillus*) possessing different bile salt tolerance and ability to resist low pH (pH 1.80) in presence of pepsin were used: probiotic strains *L*. *bulgaricus* S6, *L*. *bulgaricus* S22, *L*. *bulgaricus* S11, *L*. *bulgaricus* S10, *L*. *bulgaricus* SR, *L*. *pentosus* SS and *L*. *gasseri* S20; non-probiotic strains—*L*. *bulgaricus* S28, *L*. *bulgaricus* S8, *L*. *bulgaricus* S9, *L*. *bulgaricus* S1, *L*. *bulgaricus* Y12, *L*. *bulgaricus* S7, *L*. *bulgaricus* S4, *L*. *bulgaricus* S3, *L*. *bulgaricus* S2, *L*. *bulgaricus* S29 and *L*. *bulgaricus* S30. The strains *L*. *bulgaricus* SR and *L*. *bulgaricus* Y12 was isolated from yoghurt, *L*. *pentosus* SS was isolated from a commercial probiotic product. The rest of the strains were provided by “Selur Pharma” Ltd. (Bulgaria). All of the strains were divided later in three groups (non-probiotic, moderate and probiotic) by means of their growth rate, biomass production, minimal inhibitory concentration of bile and best recovery after 3 h at low pH and pepsin as it is described below. All microorganisms were freeze-dried and kept at -80°C.

### Preparation of stock cultures

The strains were cultivated in MRS broth (Merck, Japan) at 37°C for 24 h. The biomass obtained after centrifugation at 5000 min^-1^ for 5 min was twice washed with PBS buffer (pH 7.00) and suspended in 15%w/v glycerol solution to the initial volume and stored at -80°C for further use.

### Preparation of active bacterial culture

Tubes containing 1 ml MRS broth (Merck, Japan) were inoculated with 50 μl glycerol suspension of stock culture and cultivated for 18–20 h at 37°C.

### Determination of the optical density of the bacterial cultures

The optical density was determined by using a micro-plate reader iMark (BioRad, USA) against MRS broth as blank at λ = 665 nm. The sample volume was 150 μl with correction to 1 cm path length. Every optical density is presented as an average values of nine optical densities obtained from three independent samples (tubes or deep well plate wells) measured three consecutive times.

### Determination of maximal specific growth rates of the strains

Tubes containing 750 μl MRS broth were inoculated with 50 μl with active bacterial culture and cultivated at 37°C for 24 h and the optical density at λ = 665 nm was determined on certain time intervals. The maximal specific growth rates were calculated based on the slope of growth curves in the logarithmic phase [22].

### Determination of the resistance to low pH value in presence of pepsin

A modified method of Pitino[14] was used. 750 μl MRS-broth were inoculated with 50 μl of active bacterial culture and cultivated for 18–20 h at 37°C. The culture medium was then centrifuged at 10000 min^-1^ for 5 min, the biomass was washed twice with PBS buffer (pH 7.00). The cells were suspended to the original volume with the low pH buffer (pH 1.8), containing HCl (0.2 M), NaCl (0.08 M), CaCl_2_ (0.03 mM), and pepsin from porcine gastric mucosa (9000 U/ml)(Wako, Japan). After 3 h cultivation at 37°C the biomass were centrifuged at 10000 min^-1^ for 5 min and washed with PBS buffer and re-suspended to its original volume with PBS buffer. Tubes containing 750 μl MRS broth were inoculated with 50 μl of low-pH treated cells suspensions and cultivated at 37°C for 24 h. The optical density of the cultures was measured at λ = 665 nm at 0h and 24 h. Strains’ resistance to low pH in presence of pepsin is evaluated by cell growth and presented by the increase in the optical density of the culture medium after 24h cultivation 37°C (“Yield of biomass after 3 h stay at pH 1.80 and 9000 U/ml pepsin”).

### Determination of bile minimal inhibitory concentration

MRS broths (750 μl) with double-fold decreasing concentrations of dry bile (Wako, Japan) 0,156–5,000 mg/ml were inoculated with 50 μl active bacterial culture and cultivated at 37°C for 24 h and the optical density at λ = 665 nm was determined.

### Monitoring of the cultures by NIR Spectroscopy

MRS broth (15 ml) was inoculated with about 0.5 ml active bacterial culture to OD = 0.1 (λ = 665) and cultivated at 37°C for 24 h with shaking on vibratory shaker in a 50 ml centrifuge tube. The NIR transflectance spectra of the culture were acquired in the entire spectral region (400–2500 nm) with 0.5 nm step (4200 data points) at every 4 min by using a FOSS XDS OptiProbe Analyzer attached with immersion type probe (FOSS NIRSystems, Inc., Hoganas, Sweden or Hilleroed, Denmark, recently distributed by Metrohm NIRSystems AG, Herisau, Switzerland). Reference spectrum was taken at the beginning of every measurement series placing the immersion probe in dark aperture position of the instrument. The spectra taken in the first 40 min of the cultivation time were discarded and those after 40 min until the scan of 20 h of the monitoring were used for data evaluation. Total number of spectra in the experiment = 15 strains x 300 spectra = 4500 (S1 Dataset).

Spectra acquisition was performed with the VISON 3.50 (FOSS NIRSystems, Inc., Hoganas, Sweden) software. After pre-experiments, 0.5 mm layer thickness (set by spacer) was found to be the most appropriate to achieve applicable signal in the first overtone region of water.

### Data analyses

The wavelength range 1100–1850 nm was used for data evaluations. As a first step of spectral pretreatment, smoothing by using Savitzky-Golay[23] filter with 21 data points and second polynomial order was applied. For eliminating the scattering effect MSC (multiplicative scatter correction) transformation[24] was performed. As a scaling method, Pareto scale was used.

Principal Component Aanalysis[25] was used to discover the multidimensional pattern of variations in the NIR spectral dataset. Furthermore, Moving Window Principal Component Analyses was performed in order to find the most appropriate part of the cultivation time, where best discrimination of the strains having different properties could be obtained. The MW-PCA models were calculated using a window of 10 spectra of each strain and moving one spectrum forward for every step, calculating 290 PCA models. In addition to the visual representation of the PCA score plots, the ratio of the Euclidian distances of group centres and standard deviations (SD) of the three groups in the PCA plain was also calculated for every single time point.

Orthogonal Projection to Latent Structures Discriminant Analyses[26] (OPLS-DA) was applied to classify the three groups having different resistance to bile and low pH. The OPLS-DA models were validated using one-strain-out validation. The data set was split into training and test sets. The spectral data of 14 strains were used as training set; and those of one strain left, as the test set. This process of data splitting was repeated 15 times to ensure that the data of all the strains have the possibility to be included in the evaluation set once[27].

To find relationship between spectral data and phenotype parameters of bacterial strains (bile’s MIC and ability to recover after 3 h stay at low pH) we applied Partial Least Squares Regression[24] (PLSR). The PLSR models were evaluated by the coefficient of determination in calibration (R^2tr), root mean squared error of calibration (RMSEC), coefficient of determination in cross-validation (R^2cv) and root mean squared error of cross-validation (RMSECV). The maximum number of LVs was determined as 1/10^th^ of the number of observation (n) in order to avoid overfitting. The PLSR models were validated using the same one-strain-out validation method as we applied for the testing of the OPLS-DA models.

Aquagrams[20] were calculated in order to show the differences of the absorbance values at the water matrix coordinates (WAMACs) for the group of probiotic, non-probiotic and moderate bacteria. The star-chart displays averaged normalized spectral absorbance values of the groups of probiotic, non-probiotic, moderate strains and mQ water (acquired at the same conditions). The strains spectra are acquired at 37°C in the time interval of 11.4–12 h of the cultivation time.

The scripts for MW-PCA, PLSR and Aquagram calculation and visualization were written and executed in R-project environment (RStudio Ver. 0.98 and R Ver. 3.0.1, R Foundation for Statistical Computing, Vienna, Austria). The calculation and visualization of PCA and OPLS-DA were performed with Simca-P+ Ver. 13.5 (Umetrics AB, USA).

## Results

All of the strains included in this study represent three species of genus *Lactobacillus*. Thus, they possess similar morphological, metabolic and physiological characteristics. They ferment the glucose to lactic acid as a major end product by homolactic fermentation (Embden-Meyerhof-Parnas pathway and subsequent pyruvate reduction) and require complex growing medium containing different growth factors[28]. The strains vary in their ability to survive and grow under stress conditions as well as in their growth rate and maximal yield of biomass. This is due to their adaptation ability and depends on the presence and the expression of some genes, which leads to differences in the levels of some proteins and enzyme activities[29]. In this study we show that NIR spectroscopy and Aquaphotomics could be used successfully for finding the relationship between some phenotype characteristics of the strains and their NIR spectra.

### Analysis of the strains’ phenotype characteristics

During the culture growth, the turbidity of the culture medium increases proportionally to the cells number, which makes the optical density of the medium suitable for assessment of the cells concentration[30]. The most commonly used wavelengths for measurement of bacterial growth are in the range of 430–680 nm[31].

**Fig 1:**
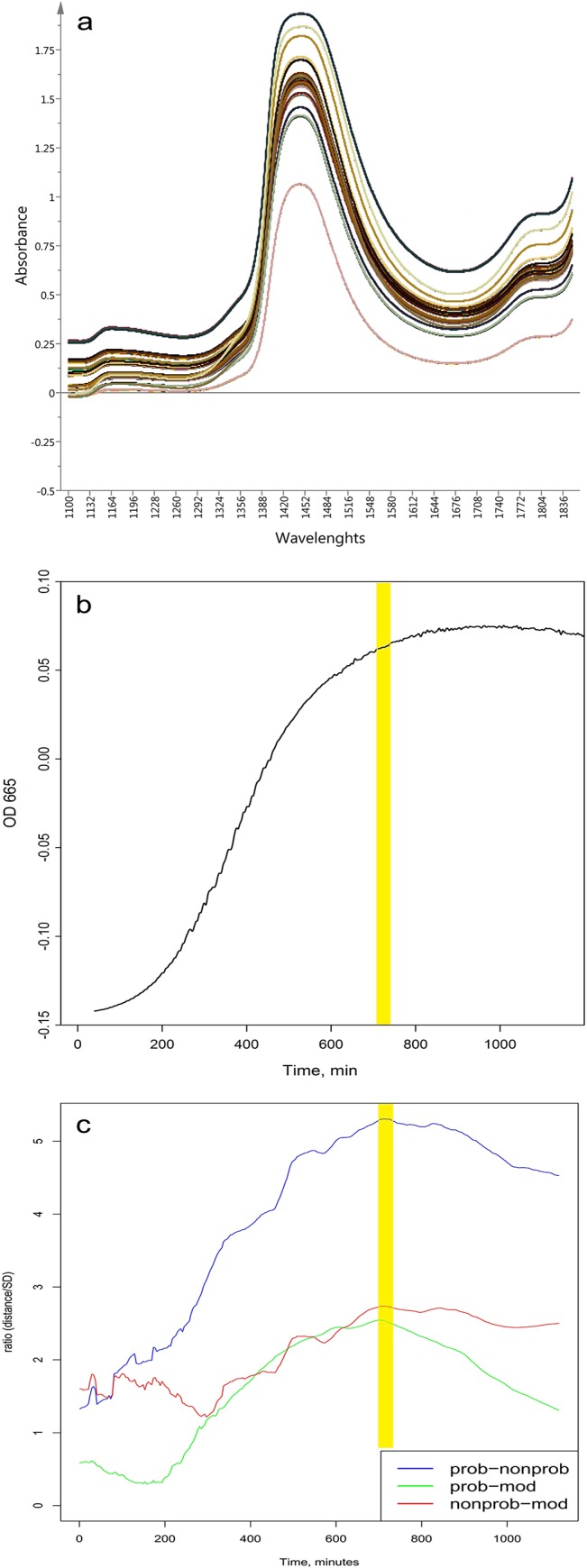
a) Truncated (1100-1850nm) raw spectra (n = 4500) of the analyzed 15 *Lactobacillus* strains acquired between 40 min and 20 h of the cultivation time; b) Growing dynamics of *L*. *bulgaricus* S6 determined at λ = 665 nm; c) Calculated ratio between the distances of group centers and standard deviations (SD) of probiotic, moderate and non-probiotic groups in the plain of PC2 and PC3 of MW-PCA calculated on the truncated (1100-1850nm) NIR data in the function of the cultivation time.

The growth rates and maximal biomass yield of the 18 *Lactobacillus* strains, as well as their viability in presence of different bile concentrations, and ability to recover after 3 h at low pH and pepsin were analyzed (Table 1 and Fig 1A.). The optical density of the culture media (at λ = 665 nm) was used as an assessment criterion for the cells concentration. The results are presented in Table 1. According to the obtained data the strains can be divided in three groups. The first group includes the strains with the highest maximal optical density and growth rate, the highest MIC (Minimal Inhibitory Concentration) of bile and best recovery after 3 h at low pH and pepsin –*L*. *bulgaricus* S6 *L*. *bulgaricus* S22, *L*. *bulgaricus* S11, *L*. *bulgaricus* S10, *L*. *gasseri* S20 and *L*. *pentosus* SS. The second group contains the strains with medium results according to these criteria (*L*. *bulgaricus* S28, *L*. *bulgaricus* S9, *L*. *bulgaricus* S1, *L*. *bulgaricus* Y12, *L*. *bulgaricus* S7, *L*. *bulgaricus* S8 and *L*. *bulgaricus* SR) and the third represents the strains with the lowest results (*L*. *bulgaricus* S4, *L*. *bulgaricus* S3, *L*. *bulgaricus* S2, *L*. *bulgaricus* S29 and *L*. *bulgaricus* S30). These three groups were used for further analysis of the correlation between their NIR spectra and their probiotic potential. Strains *L*. *pentosus* SS, *L*. *bulgaricus* S8 and *L*. *bulgaricus* SR were further used for independent validation of OPLS-DA and PLSR models and their spectra were not included in the models dataset.

**Table 1:** Growth rates, maximal optical densities, bile’s MICs and yields of biomass after low pH stress in presence of pepsin of the strains.

Strain’s group	Strains	Maximal growth rate (μ_max)_ in MRS broth, h^-1^	Maximal optical density (λ = 665nm) in MRS broth	MIC of Bile, mg/ml	Yield of biomass after 3 h stay at pH 1.80 and 9000 U/ml pepsin
Probiotic strains	S10	0.115±0.012*	2.950±0.075	2.500	0.126±0.014
	S11	0.118±0.013	2.950±0.066	2.500	0.100±0.014
	S20	0.304±0.011	3.030±0.063	2.500	0.114±0.011
	S22	0.106±0.012	2.677±0.092	2.500	0.117±0.016
	S06	0.301±0.012	2.960±0.075	1.250	0.080±0.010
Moderate strains	S01	0.121±0.011	1.692±0.058	0.625	0.038±0.010
	S07	0.075±0.012	1.919±0.068	0.625	0.049±0.013
	S28	0.106±0.024	2.770±0.087	0.313	0.025±0.006
	S09	0.118±0.017	2.880±0.081	0.156	0.029±0.005
	Y12	0.150±0.027	2.023±0.074	0.625	0.041±0.006
Non-probiotic strains	S02	0.070±0.011	1.521±0.035	0.156	0.006±0.001
	S03	0.060±0.009	1.343±0.039	0.313	0.005±0.002
	S04	0.060±0.012	0.841±0.046	0.156	0.007±0.002
	S29	0.060±0.010	1.440±0.046	0.625	0.005±0.002
	S30	0.050±0.011	1.360±0.065	0.625	0.006±0.002
Moderate	S08	0.080±0.010	2.940±0.093	0.625	0.049±0.007
Probiotic	SR	0.070±0.012	1.676±0.046	1.250	0.036±0.004
Probiotic	SS	0.220±0.024	2.963±0.085	2.250	0.107±0.010

*—Standard deviations calculated based on three parallel samples scanned three consecutive times.

**Fig 2:**
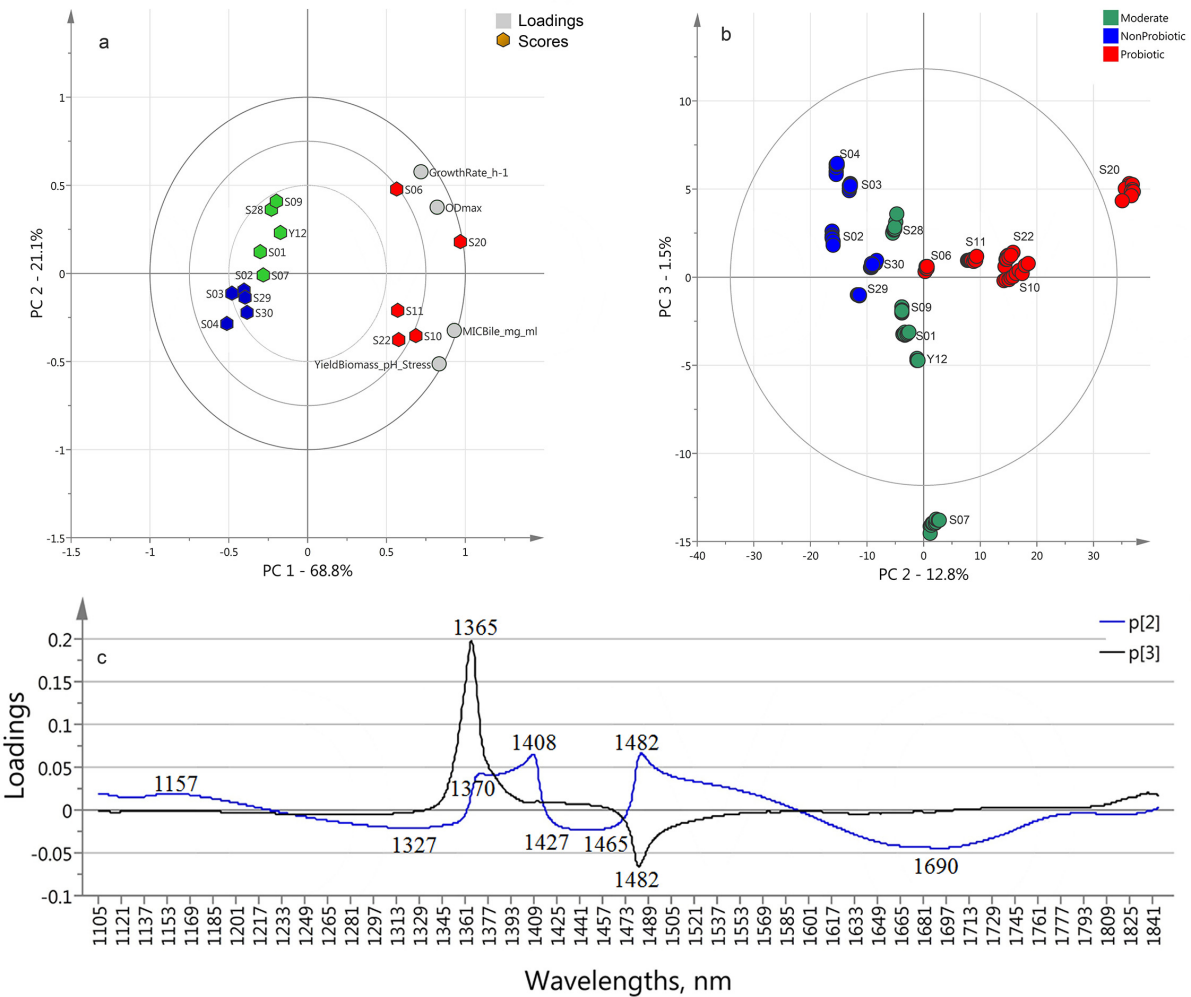
a) PCA Bi-plot calculated on the reference data (strains growth rates, maximal optical densities, bile MIC and the yield of biomass after three hours stay at pH 1.80 in presence of pepsin (9000 U/ml), reference data in Table 1); b) MW-PCA analyses using the 1100–1850 nm wavelength interval—Score plot calculated on spectral data (n = 150) at the cultivation time of 11.4–12 h. Probiotic (red symbols), moderate (green symbols) and non-probiotic (blue symbols) groups; c) loadings of PC2 (blue line) and PC3 (black line) of MW-PCA model highlighting the bands.

For the first time, all the biochemical reference data obtained when analyzing the strains were subjected to PCA (Principal Component Analysis) in order to obtain a general parameter to express probioticity, which can explain the ability to grow and survive through human gastro-intestinal tract and to sustain their viability, which is essential for expressing their probiotic action. This probioticity parameter could be used as a complex parameter for quality assessment of the probiotic strains. As an input data, strains’ growth rate, maximal optical density, bile tolerance and pH resistance were used in order to calculate PCA (reference-based PCA) (Fig 2A.) scores. The first principal component (PC1) of this matrix explains 68.8% of the total variance. Its scores are highly correlated with strain’ probiotic properties and presents very well the ability of the strains to grow in presence of bile and to survive at very low pH environment, as well as their maximal growth rates and biomass production. For the first time in this study, the reference data were analyzed using PCA and the scores of the PC1 were used as a single probioticity parameter generalizing strains resistance to environment similar to the conditions in human gastro-intestinal tract, as well as their maximal growing rates and ability to produce biomass.

### Determination of the most appropriate cultivation time for probiotic strain identification when using spectral data analysis

The NIR spectral characteristics of the strains change during their cultivation process. In order to identify each strain and evaluate its probioticity using only its spectral monitoring data, we analyzed the spectral data (S1 Dataset) to select the most appropriate time window for further data analysis. According to phenotype analysis, for adequate comparison of the strains they should be in the same growth phase. This phase should be the one that gives NIR spectra with the most significant differences between the groups of strains. At the same time, the differences between the strains within the groups of strains with the same phenotypic characteristics should be minimal. To determine the most appropriate time that meets these requirements a “Moving window PCA” (MW-PCA) calculations on spectral data were performed using R-project software. The results of spectral analysis showed that there were smaller differences between the groups of strains in the beginning and at the end of the cultivation process. Consistently with the analysis of the strains phenotype, the most significant differences between the groups were observed at the end of the exponential growth phase. Also, in this phase the strains within one group showed minimal differences between each other. This resemblance is due to the large number of similar cells, which are still viable. Due to a strong influence of the temperature, seen in the loading plot of the PC1 (not shown), the best separation of the three groups were based on PC2 and PC3 (Fig 2B.) scores. Therefore, the calculation of the quotient for distance and SD was performed based on the scores of PC2 and PC3. The calculated ratio confirmed the observations of the visual evaluation of the PCA score plots (Fig 1C.). The optimal time for the best separation of the three main groups was found to be when the distance between two group centers is the highest and at the same time the standard deviations of the groups are the lowest for all the three pairwise cases (probiotic – moderate, probiotic – non-probiotic and non-probiotic – moderate). On the basis of the results of MW-PCA, the most appropriate time for data analyses was set to be 11.4–12 h of the cultivation process (Fig 1B).

### Discrimination of probiotic strains based on their growth monitoring spectral data

Strains growth monitoring spectral data acquired at the time interval of 11.4–12 h has been analyzed with MW-PCA. The PCA score plot calculated on the spectral data of the strains (NIR-based PCA) at the time period of 11.4–12 h of the cultivation time is shown in Fig 2B. The projection of PC2 and PC3 plane of NIR-based PCA showed the biggest similarities to the reference-based PCA results (Fig 2A and 2B., respectively). There is no distinct separation of the three groups on the PCA plane. The second component which presents 12.8% of the total variance shows that the spectra of the moderate group are placed in the center of the plot and the other two groups are on the left (non-probiotic) and on the right (probiotic).

The loadings of PC2 and PC3 for the NIR-based PCA showed peaks in the entire spectral range, but the most important bands were in the range of 1300-1600nm, the first overtone of water. The wavelengths responsible for the separation of the three main groups of the strains are at 1157, 1327, 1365, 1370, 1408, 1482 and 1690 nm.

OPLS-DA (Orthogonal Projection to Latent Structures Discriminant Analyses) method could be applied for classification of biochemical data, which in many cases is multi-collinear and noisy. This is a powerful technique which combines the strength of PLS-DA (Partial Least Squires Discriminant Analysis) and SIMCA classification methods[26]. It uses reduced numbers of discriminant functions, which makes easier the interpretation of observed discriminations.

**Fig 3:**
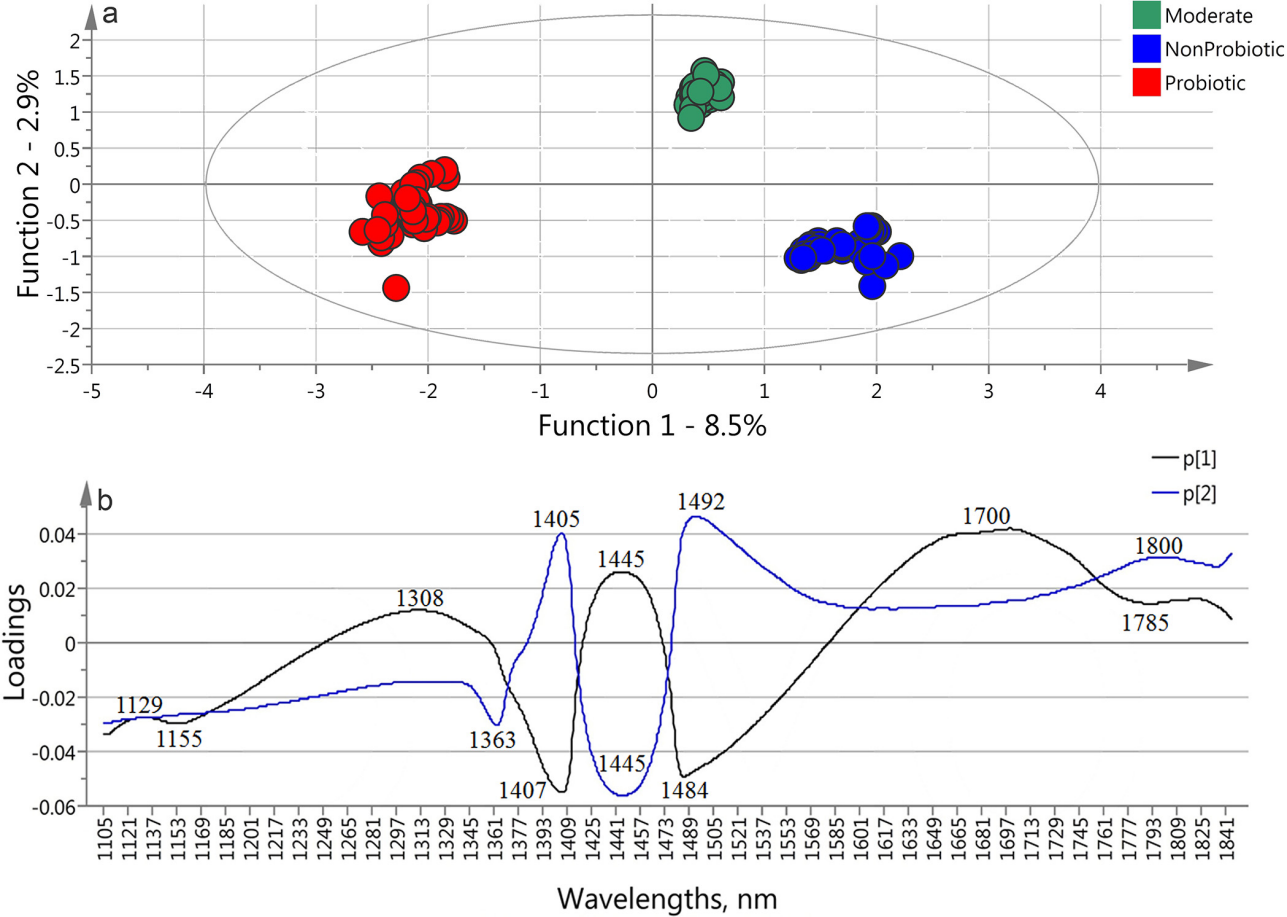
OPLS-DA model built on the spectral data of the 15 strains in the monitoring time between 11.4–12 h (n = 150) using the 1100–1850 nm wavelength interval to classify the probiotic, moderate and non-probiotic groups a) score plot and b) loadings plots.

With our spectral data set OPLS-DA method provided a clear separation of the three main groups. The score plot of the first two functions (Fig 3A) shows very distinct groups of the data points representing the strains having different characteristics. The first discriminant function containing 8.5% of groups’ variance of the spectral data provides the best separation between the probiotic and non-probiotic groups. The second discriminant function (2.9%) is responsible for the discrimination of the moderate group from the above mentioned ones. The results of classification matrix of the cross-validation (one strain out) process showed 100% correct classification and recognition of the strains’ groups, which confirm the robustness of the model.

In order to test the model’s potential for classification of new strains, independent validation was performed. Every strain is presented by its ten spectra, which were excluded of the model consecutively and used as an “unknown strain data”. On the base of the rest of 14 strains were built 15 different OPLS-DA models for prediction of the “unknown strain” sets, so that every strain was tested with the model where its spectra were excluded. The results show no misclassification between probiotic and non-probiotic groups. Three strains were misclassified – the “weakest” probiotic and moderate strains (*L*. *bulgaricus* S6 and *L*. *bulgaricus* S28) were classifies as a moderate and non-probiotic strains, respectively, and the moderate strain *L*. *bulgaricus* S1 was classified as non-probiotic. *L*. *bulgaricus* S6 and *L*. *bulgaricus* S28 are on the border of their groups, which explains the incorrect classification. Correctly classified strains present 80% of the total number included in the experiment. Three new strains, presented by their 10 spectra, acquired at the same time interval were used to test the generalization of the model. Their spectra were used as test sets for classification of those “new strains.” The strains were put in the model one by one and were classified with high accuracy. All of *L*. *bulgaricus* S8 and *L*. *pentosus* SS spectra were classified correctly as moderate and probiotic respectively. The spectra of *L*. *bulgaricus* SR were classified as probiotic – 70% and non-probiotic – 30%.

These results could be explained with the fact that classification based on spectral data includes much more molecular information about the solute and the solution than the few initial biochemical parameters.

The loadings of the first two discriminant functions of OPLS-DA model are shown in Fig 3B. The peaks found at 1155, 1363, 1405, 1407, 1484 and 1700 nm appeared in similar wavelength ranges (with several nm shifts) at the MW-PCA loading vectors. They show consistently high importance of these particular bands for the separation of the three groups using OPLS-DA method.

### Quantitative prediction of the strains’ growth resistance to low pH and bile when using strains growth monitoring spectral data

**Fig 4:**
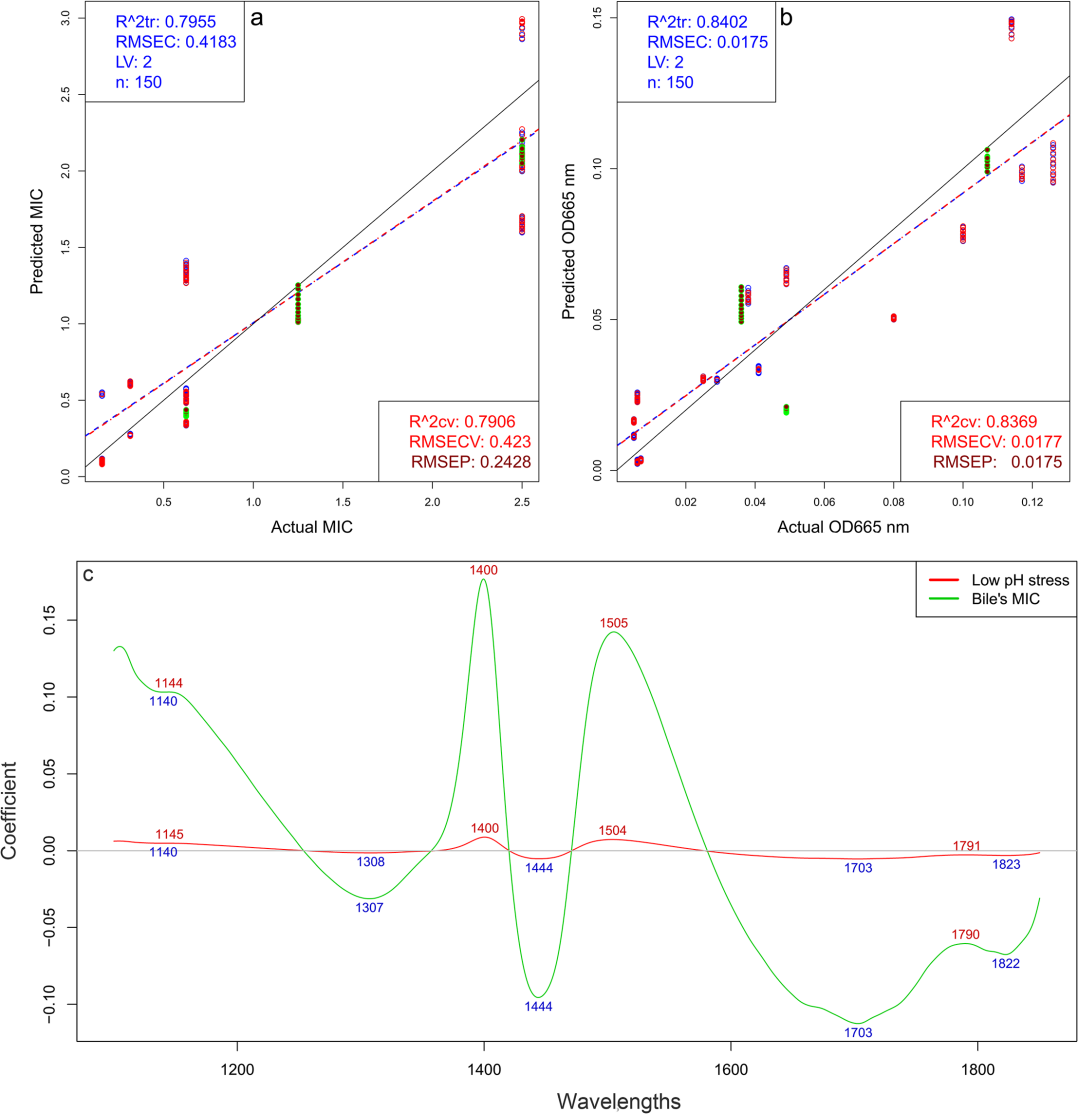
PLSR models on spectral data obtained between 11.4–12 h (n = 150) of the cultivation process, wavelength interval 1100–1850 nm a) Quantified MICs vs. NIR-predicted MICs of bile tolerance of the *Lactobacillus* strains. Calibration (blue line and points), “one strain out” cross-validation model (red line and points) and strains not included in the modeling dataset (green dots); b) Quantified optical densities at 665 nm vs. NIR-predicted optical densities for strains cultivated in MRS after 3 h treatment at low pH and pepsin, c) PLS regression vectors, indicating the bands of biggest importance for the discrimination.

Regression models were built to determine relationship between spectral data and optical densities at 665nm after 3 h treatment of the *Lactobacillus* strains at low pH and pepsin (Fig 4B) and MICs of bile of the *Lactobacillus* strains (Fig 4A). Results of PLSR (Partial Least Squares Regression) models show close correlation and relatively low error of calibration and cross-validation using only two latent variables.

The results of the PLSR models are presented in Fig 4. The regression models show close correlation in models building and in “one strain out” cross-validation process. During cross-validation procedure, the data of one strain were left out of the training set and were used as test set, then data of another strain were left out iteratively, until all strains were used for test at once. Relatively low error of prediction (RMSEP) was found during the calibration and cross-validation of the model, using only two latent variables.

Independent validation of these models was performed by using three strains which spectra were not included in the models’ dataset—*L*. *pentosus* SS, *L*. *bulgaricus* S8 and *L*. *bulgaricus* SR. Their resistance to low pH and bile was predicted by the models with high accuracy and low error of prediction (Fig 4A and 4B) RMSEP values of these strains are 0.2902 for *L*. *bulgaricus* S8, 0.0190 for *L*. *bulgaricus* SR and 0.004 for *L*. *pentosus* SS when were predicted their low pH resistance, and 0.2130 for *L*. *bulgaricus* S8, 0.1481 for *L*. *bulgaricus* SR and 0.3671 for *L*. *pentosus* SS after the prediction of their bile MIC. All values are in the range of 3–16% of the total calibration ranges with the exception RMSEP of *L*. *bulgaricus* S8 low pH tolerance, which is 24%.

The main absorbance bands showing significant weight in the PLS regression vector (Fig 4C) match very well with the bands found in the previously applied methods (Fig 2C, Fig 3B and Fig 4C) It is another confirmation of the importance of the spectral range of the first water overtone (1300-1600nm). Therefore the information described by the first overtone range of water gives the opportunity to build a highly accurate model to predict strains ability to grow and survive conditions similar to those in human upper gastrointestinal tract. In other words, we discovered that the spectral pattern of the water molecular system presented by its covalent and hydrogen bonds and measured in the NIR region could be used as a holistic biomarker highly related to the functionality of the whole system of each strain.

Another successfully applied approach to examine the first spectral overtone of water proposes twelve specific spectral ranges which are of biggest importance. The “Aquagram” is a star-chart which contains normalized absorbance values at wavelengths in those regions of interest. These values contain information about water molecular conformations and their respective hydrogen and covalent bonds[16,20].

**Fig 5:**
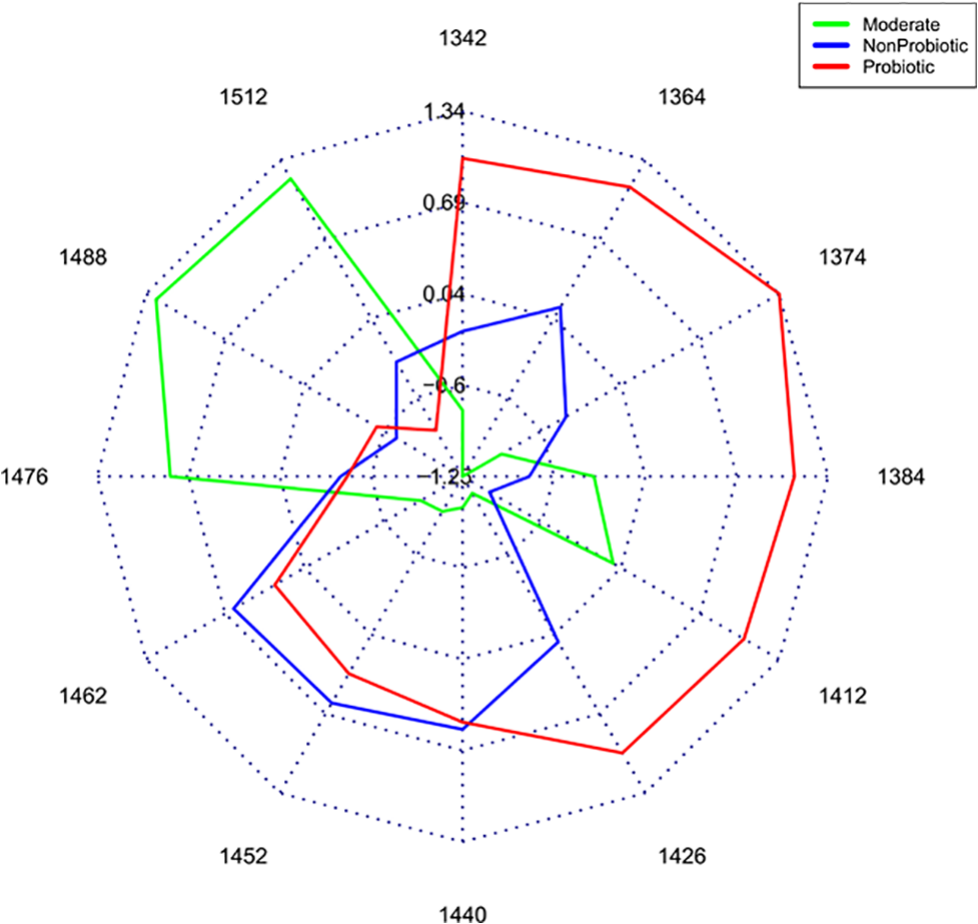
Aquagram on the spectra of culture media of groups of probiotic, moderate and non-probiotic strains. Averaged values of normalized absorbance values of the water matrix coordinates for every group are plotted on each axis. Results were calculated on spectral data obtained between 11.4–12 h.

In the aquagram (Fig 5) the three groups showed biggest differences in the absorbance values in the region of 1365–1426 nm, where the group of probiotic bacteria have biggest absorbance. In the region of 1440–1462 nm the groups of probiotic and non-probiotic bacteria show similar patterns. The group of the strains with moderate scores (Table 1) absorb the best in the region of 1476–1512 nm.

The wavelengths presented in the aquagram are characteristic of protonated water molecules presented by the asymmetric OH-stretch vibrational frequencies of [H^+^·(H_2_O)_3_], [H^+^·(H_2_O)_4,5_] and [H^+^·(H_2_O)_6,7_] water clusters with different size (at 1342, 1374 and 1486 nm respectively); water shell OH stretch of water clusters with different size—[OH^-^ ·(H_2_O)_2_], [OH^-^ ·(H_2_O)_4_] and [OH-·(H_2_O)_5_] at 1364, 1440 and 1452 nm respectively; H_2_O-OH bonded water molecules at 1384 nm; free water molecules (S_0_) at 1412 nm; water molecules bound to protein (protein hydration) at 1426 nm and S_2_ ((H_2_O)_3_) and S_3_ ((H_2_O)_4_) water clusters at 1462 and 1476 nm [32–35].

## Discussion

The selection of strains possessing probiotic properties has been done using different approaches. Many authors approach is based on isolation of big number of strains and *in-vivo* evaluation of their capability to survive in simulated gastrointestinal tract conditions, in presence of different antibiotics and other antimicrobial substances, their antimicrobial activity and their ability of adherence to human cells lines [36–38]. Others focus on studying of particular genes expression and genome DNA profiling of the studied bacteria [39]. Both approaches are time-consuming and require complicated sample preparation. In this paper we present a new technology and concept, which demonstrate that NIR spectroscopy and Aquaphotomics when applied for differentiation of closely related microorganisms with different phenotypic characteristics provide very accurate, fast and non-invasive identification of probiotic strains based on spectral monitoring data of bacterial growth at 11.4–12 h of cultivation time. For the first time, this method was used for *in-vivo* evaluation of probiotic and non-probiotic lactobacilli.

The multivariate methods applied for the spectral data assessment in regards to phenotype identification showed several common absorbance bands, with high importance, i.e. weight in the models of strains identification and parameters quantification. The wavelengths significant for the classification of probiotic strains and those which are responsible for the prediction of their survival rate are summarized in Table 2. The highlighted bands were based on our experimental spectral data and were found statistically when applying PCA, OPLS-DA and PLSR methods. Most of the bands with high variations of their absorbance were consistent with the described 12 water matrix coordinates (WAMACS) [16] described for the first overtone of water. Among these wavelengths is 1386, which is in the region 1370–1408 and 1700 corresponding to higher protonated water clusters [32,34]. The bands at 1484 and 1492 correspond to the first order stretching overtone of O–H-O and the first overtone of the highly hydrogen bonded S_4_, ((H_2_O)_4_), water cluster, respectively. In the OPLS-DA model we found a characteristic band with maximum at 1155 nm which corresponds to the combination overtone of the free water molecules (S_0_)(unpublished data). Similar picks (at 1157 and 1144 nm) appear in the same region in PCA loadings and PLSR regression vectors, respectively (Figs 2 and 3).

**Table 2:** Measured wavelength and calculated wavenumbers of the bands found with PCA, SIMCA, OPLS-DA and PLSR methods and their assignment based on the corresponding references.

Measured wave-length (nm)	Calculated wave-number (cm^−1^)	Calculated fundamental wavenumber (cm^−1^)	Assignment	Ref
1155		-	Combination overtone of free water (S_0_)	-
1365	7326	7326/2 = 3663	OH, 1st overtone, aqueous proton [H+·(H_2_O)_2_]—H_2_O asymmetric stretch	[40]
			OH, 1st overtone, Dangling-OH (non-hydrogen-bonded	[41]
			OH, 1st overtone, H_2_O v1	[42]
			OH, 1st overtone, H_15_O_7_+	[43]
1386	7215	7215/2 = 3607.5	OH, 1st overtone, Superoxide Tetrahydrate O_2_-.(H_2_O)_4_	[44]
			OH, 1st overtone, H+(H_2_O)_10_	[40]
			C–H stretching, sucrose	[45]
			OH, 1st overtone, OH^-^ stretching mode	[46]
1408	7100	7100/2 = 3550	OH, 1st overtone, H-bonded OH stretch	[47]
			O–H, 1st overtone, glucose bonds	[48]
			OH, 1st overtone, OH stretching in alcohols	[49]
			OH, 1st overtone, hydrogen-bonded dimers	[50]
1450	6895	6895/2 = 3447.5	OH, 1st overtone, deionized water	[51]
			OH, 1st overtone, O-H stretch	[52]
			combination of antisymmetric and symmetric stretching modes of water	[53]
1485	6735	6735/2 = 3367.5	OH, 1st overtone, H_17_O_8_+	[43]
			OH, 1st overtone, H_15_O_7_+ H-bonded OH stretch	[47]
			NH, 1st overtone, amid	[54]
			NH/OH, 1st overtone, N–H/O–H stretching	[55]
1492	6700	6700/2 = 3350	OH, 1st overtone, hydrogen-bonded (S_4_)	[34]
			OH, 1st overtone, H_15_O_7_+	[43]
			OH, 1st overtone, strongly H-bonded	[56]
			NH, 1st overtone, N–H stretching	[57,58]
			NH, 1st overtone, NH_2_'s asymmetric stretch	[59]
1698	5890	5890/2 = 2945	OH 1st overtone, Superoxide Tetrahydrate O_2_-.(H_2_O)_4_	[44]
			C−H vibration	[60]
			CH/CH2 combination band	[61]
			H–O–H/O–H bending and translation/rotation combinations	[62]
1819	5500	5500/2 = 2750	1st overtone IHB stretch (OH-(H_2_O)_3_)	[63]
			combinationν(C−H) + ν(O−D)free	[64]

The bands found in our models are mainly due to the presence of free water molecules, water solvation shells, protonated water and other water molecular conformations. From our results (Fig 5), statistically, we found that the group of probiotic bacteria characterises with higher number of small protonated water clusters, free water molecules and water clusters with weak hydrogen bonds in comparison with the other two groups. In contrast, the moderate group shows large number of bigger water clusters with strong hydrogen bonds. The group of probiotic bacteria also show big absorption in the region of water-protein interactions followed by the moderate and non-probiotic strains. There are also bands of different functional groups of the main biopolymers building the living cell. In this paper, having in mind the big difference in concentration when comparing with water, we have focussed mainly on the water specific absorbance bands. We presume that the rest of the molecules in the media influence and coordinate the surrounding water molecular matrix and lead to changes in the water bands, i.e. water behaves as molecular mirror. These bands show the importance of the cells compounds for the classification and the prediction of the strains phenotype. This could be due to the differences in the levels of many hydrated organic components and differences of water molecular conformations inside and outside the cells. Thus, the information provided by the water conformation reveals the important differences between probiotic and non-probiotic *Lactobacillus* strains.

The NIR spectral analyses allowed highly accurate qualitative and quantitative analysis of bacteria. Both of them reveal the importance of the first overtone spectral range of water (1300–1600 nm) as molecular system. Water spectral patterns were successfully used as biomarkers leading to highly accurate and fast classification and prediction of the different phenotypic properties of potential probiotic candidates of genus *Lactobacillus*. These results demonstrate the potential for application of Aquaphotomics as rapid holistic approach in the screening and evaluation of probiotic microorganisms and their functionality.

## Supporting Information

S1 Dataset NIR transflectance spectra of all strains, acquired in the entire spectral region (400–2500 nm) with 0.5 nm step at every 4 min. (RAR)
